# Pancreas herniation into the mediastinum: a case report

**DOI:** 10.1186/s13104-017-2799-y

**Published:** 2017-09-06

**Authors:** Muhammad Shafiq, Moaviz B. Badshah, Maaz B. Badshah, Mashood B. Badshah, James Watkins

**Affiliations:** 1 Department of Internal Medicine, University of Missouri, Kansas City (UMKC), 2411 Holmes Street, Kansas City, MO 64108 USA; 2 Internal Medicine, Jamaica Hospital Medical Center, New York, USA; 3 University of Illinois at Peoria, Peoria, Illinois USA; 4 Khyber Medical College, Peshawar, KPK Pakistan; 5 Indiana University School of Medicine, Indianapolis, IN USA

**Keywords:** Mediastinum, Herniation, Acute pancreatitis, Abdominal pain, Ectopic pancreas

## Abstract

**Background:**

Pancreatic tissue found in the mediastinum (both true ectopic and herniated pancreas) is rare. It becomes even more challenging when there are complications associated with this entity.

**Case presentation:**

We report an unusual case of pancreatic herniation into the mediastinum in a 90-year-old Caucasian female. This patient initially presented with nausea and vomiting associated with abdominal pain. Serum lipase and amylase both were elevated. Computed tomography scan of the chest, abdomen and pelvis revealed a large hiatal hernia with pancreas herniation into the mediastinum, with superimposed acute pancreatitis likely due to gallstone. Because of its unusual location, the patient also developed acute mediastinitis. The patient was management conservatively and did well. On the day of discharge; she was tolerating a diet, had no pain or nausea and was back to her baseline health.

**Conclusion:**

Acute pancreatitis can be managed conservatively even if it is in the mediastinum. Also, ectopic or herniated pancreatic tissue is extremely rare and leads to unique clinical presentations, along with diagnostic and therapeutic challenges. Clinicians should not only be vigilant to the presence of ectopic or herniated pancreatic tissue, but also be mindful of the resulting complications.

## Background

It is uncommon for the pancreas to be present other than at its expected location in the retroperitoneum. This is documented in 0.6–13% of autopsies [1]. Unusual Locations for pancreatic tissue include stomach [2], duodenum [3], jejunum [4] and colon [5]. They are usually asymptomatic [6]. It is very rare for pancreatic tissue to be found in the mediastinum. Complications arising at these unusual locations are rarely reported and pose unique diagnostic and therapeutic challenges [7, 8].

## Case presentation

Patient is a 90-year-old Caucasian female with a past medical history of gastroesophageal reflux disease symptomatically managed with pantoprazole 40 mg daily, hypothyroidism, depression, anxiety, and peripheral neuropath; who presented to the emergency department (ED) with nausea, vomiting and abdominal pain. She also had a fever of 101.5 F and was complaining of chills. She initially went to a local ED where laboratory evaluation revealed a urinary tract infection. She was found to have elevated serum amylase (890 U/L) and lipase (3500 U/L) levels as well. Her white blood cell count was 11,000/mcL. Total bilirubin, alanine aminotransferase and aspartate aminotransferase levels were normal. Her blood cultures grew Gram-negative rods and Gram-positive cocci for which she was started on vancomycin [1250 mg every 12 h] and Zosyn^®^ [3.375 gm every 6 h].

A computed tomography (CT) scan of the abdomen and pelvis without intravenous contrast was performed which showed a very large hiatal hernia compressing and deforming the heart amongst other structures. The majority of the stomach along with the body and tail of the pancreas were herniated into the mediastinum. There was a large amount of free fluid in the mediastinum. There was dependent cholelithiasis and a distended gallbladder with signs of acute cholecystitis. A right upper quadrant ultrasound (RUQ US) showed intra and extra-hepatic biliary ductal dilation with possible echogenic material within the common bile duct. There was gallbladder wall thickening and peri-cholecystic fluid, these findings were thought to represent either acute cholecystitis or secondary to pancreatitis. The patient underwent an echocardiogram which showed left ventricular ejection fraction of 74%. There were no regional wall motion abnormalities.

Based on the findings of RUQ US, an endoscopic retrograde cholangiopancreatogram (ERCP) was performed which showed a normal esophagus, large hiatal hernia with most of the stomach above the diaphragm and a duodenal diverticulum. The major papilla was located entirely within the diverticulum. The entire main bile duct was dilated. Choledocholithiasis was found.

**Fig. 1:**
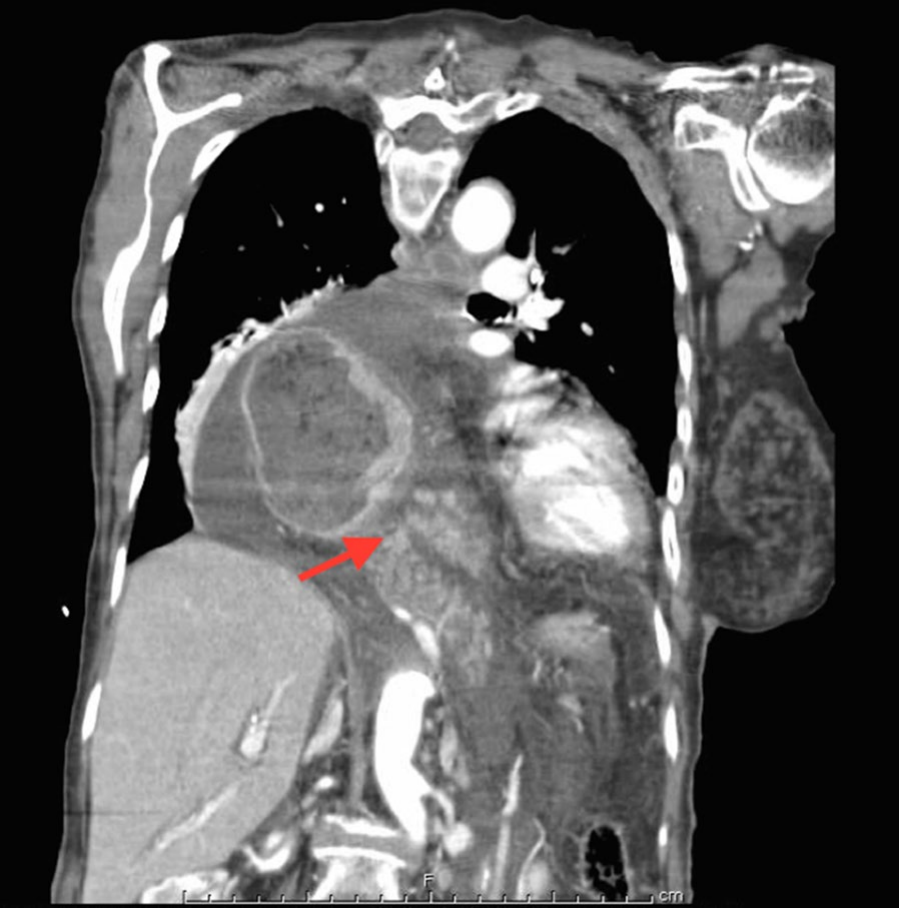
Coronal section: CT scan of the chest and abdomen, which shows the herniation of the pancreas (body and tail) into the pancreas (*arrow*)

A repeat CT scan of the chest, abdomen and pelvis with intravenous contrast was performed. The body and tail of the pancreas was found to be herniating into the mediastinum (Fig. 1).

The patient was continued on vancomycin and Zosyn^®^. The bacteria were found to be *ampicillin*-*sensitive Enterococcus durans* and a *pan*-*sensitive E. coli*. She was switched to ampicillin [2 g every 6 h] for the next 2 days and finally to oral amoxicillin PO [500 mg every 8 h]. Given the findings of large hiatal hernia, General Surgery was consulted. Given the chronicity of the patient’s symptoms, her age and relatively good symptom control with proton pump inhibitors, a conservative approach to therapy was recommended with no surgical consideration for hiatal hernia repair. An elective cholecystectomy was offered to the patient and she elected to discuss this at a clinic visit in the upcoming weeks. On the day of discharge, the patient was tolerating a diet, had no pain or nausea, and was feeling well.

## Discussion

Elevated serum amylase and lipase are sensitive and specific indicators for acute pancreatitis and should always alert the clinician [9]. After a careful history, physical examination and laboratory evaluation, CT scan of the abdomen and pelvis is usually pursued. However, if imaging does not reveal the location of pancreas in its usual retroperitoneal location, a search for pancreatic tissue in other locations should be pursued. 45% cases of pancreatic ectopic tissue occur in the stomach and duodenum, while 35% occur in the jejunum [1]. Other very rare locations include gallbladder [10], mediastinum and even fallopian tube [7, 8, 11]. Commonly used diagnostic modalities for ectopic pancreas include CT scan and endoscopic ultrasound (for upper gastro-intestinal tract).

It is very rare for pancreatic tissue to be found in the mediastinum. A complication arising in this location poses unique challenges in diagnosis and treatment for clinicians. Asymptomatic and benign ectopic pancreatic tissues are left alone without therapy [10]. Once ectopic or herniated pancreatic tissue becomes symptomatic, the presentation and treatment then depends upon its location and type of complication. Various complications include pseudo-cyst formation [4, 12], gastrointestinal tract bleeding [13], malignant degeneration and transformation [14] and rarely, gastrointestinal tract obstruction [15]. Each complication is then treated accordingly.

Our patient developed acute mediastinitis and had a very large hiatal hernia. Given this patient’s age, the risks of a surgical intervention far outweighed any potential benefits. She was therefore treated conservatively with intravenous fluids, broad spectrum antibiotics and symptomatic pain control. Fortunately, the patient recovered with these conservative resuscitative measures without any complications.

Based on our patient and a few cases that have been reported in the past [7, 8], we can deduce that chest is also a potential site for the pancreas (It can be either a true ectopic pancreatic tissue or it can be a herniation).

## Conclusion

Acute pancreatitis can be managed conservatively even if it is in the mediastinum. Also, ectopic or herniated pancreatic tissue is extremely rare and leads to unique clinical presentations, along with diagnostic and therapeutic challenges. Clinicians should not only be vigilant to the presence of ectopic or herniated pancreatic tissue, but also be mindful of the resulting complications. A case by case approach to management is appropriate in most instances.

## Abbreviations

ED: Emergency Department
CT Scan: computed tomography scan
RUQ US: right upper quadrant ultrasound
